# A Framework for Reproducible Latent Fingerprint Enhancements

**DOI:** 10.6028/jres.119.006

**Published:** 2014-04-25

**Authors:** Alfred S. Carasso

**Affiliations:** National Institute of Standards and Technology, Gaithersburg, MD 20899

**Keywords:** dodge and burn, fractional diffusion equation, latent fingerprints, progressive Lévy stable smoothing, reproducible image enhancement

## Abstract

Photoshop processing^1^ of latent fingerprints is the preferred methodology among law enforcement forensic experts, but that appproach is not fully reproducible and may lead to questionable enhancements. Alternative, independent, fully reproducible enhancements, using IDL Histogram Equalization and IDL Adaptive Histogram Equalization, can produce better-defined ridge structures, along with considerable background information. Applying a systematic *slow motion* smoothing procedure to such IDL enhancements, based on the rapid FFT solution of a Lévy stable fractional diffusion equation, can attenuate background detail while preserving ridge information. The resulting smoothed latent print enhancements are comparable to, but distinct from, forensic Photoshop images suitable for input into *automated fingerprint identification systems*, (AFIS). In addition, this progressive smoothing procedure can be *reexamined* by displaying the suite of progressively smoother IDL images. That suite can be stored, providing an audit trail that allows monitoring for possible loss of useful information, in transit to the user-selected optimal image. Such independent and fully reproducible enhancements provide a valuable frame of reference that may be helpful in informing, complementing, and possibly validating the forensic Photoshop methodology.

## 1. Introduction

This report is a sequel to [1] and a contribution to the research project *Metrics for Manipulation and Enhancement of Forensic Images*, sponsored by a 2012 NIST Forensic Measurement Challenges award. Here, an effective smoothing technique previously used succesfully in nanoscale imaging [2], is applied to gradually attenuate background detail in certain kinds of latent fingerprint enhancements discussed in [1]. This smoothing process is accompanied by an audit trail. The aim of this work is to develop independent, systematic, and easily reproducible alternatives to forensic Photoshop processing that can yield fingerprint enhancements of comparable quality. Viewed constructively, such alternative approaches provide a valuable frame of reference that may be helpful in informing, complementing, and possibly validating the Photoshop methodology.

In recent years, the reliability of fingerprint evidence has come under increased scrutiny, [3–8]. One area of concern involves the digital enhancement of latent fingerprints left unintentionally at a crime scene. Such prints are generally smudgy, with poorly defined ridge impressions that may be partially obscured by textured background structures, together with random noise. There may also be overlapping prints. The difficulty of isolating ridge impressions from such backgrounds, a key step in latent print enhancements, has been stressed by several authors, [9–11]. In [8, Chapter 4], the possibility of misleading artifacts in digital enhancements is discussed, with guidelines proposed for the proper evaluation and acceptance of enhanced images. These guidelines include validation of the enhancement technology, along with maintenance of an audit trail of the actual digital procedures used.

Photoshop processing of latent fingerprints is the preferred methodology among law enforcement forensic analysts. A variety of ‘dodge and burn’ and ‘brush’ tools are freely applied to lighten or darken selected areas within the image, and remove unwanted background information. The multiplicity and diversity of the individual steps used in this process are not fully documented, and the procedure may not be reproducible. For these reasons, forensic Photoshop latent fingerprint enhancements have occasionally been found questionable.

## 2. Behavior in Alternative Latent Print Enhancements

In [1], several fingerprint enhancement approaches independent of Photoshop are discussed, along with the software routines necessary to implement them. All of these methods aim at bringing into better visual range potentially significant structures that are present in the original image, but not easily discernible. Each of these enhancement methods is based on the use of a *single* command from such widely used scientific image analysis packages as MATLAB [12], IDL [13], and PV-WAVE [14]. Such enhancement commands are applied to the whole image at once, are executed in seconds, and are obviously reproducible. In addition, [15] provides a thorough discussion of the theoretical ideas underlying these various techniques. All images appearing in this report, and expected as input or returned as output in the software routines, are 8 bit grey scale tiff images.

While more generally applicable software is discussed in [1], we now list for the reader’s convenience a simple routine, ‘IDLHist.pro’, for performing either histogram equalization, or adaptive histogram equalization. These two distinct IDL techniques exhibit better-defined ridge structure, along with considerably more background detail than is generally the case in forensic Photoshop enhancements, and they are the primary focus of the present study. The routine below is to be applied to an 8 bit grey scale original latent print image with white ridge impressions. The routine returns the enhanced image with white ridge impressions, as well as the reversed enhanced image with black ridge impressions.


;pro-file IDLHist.pro
;SIMPLE IDL CODE FOR ENHANCING IMAGES
;SELECT EITHER Simple or Adaptive histogram equalization
;APPLY BY TYPING ‘.run IDLHist’ in IDL
;ASKS USER TO PROVIDE INPUT 8bit GREYSCALE TIFF IMAGE
;ASKS USER TO SPECIFY X size and Y size of INPUT TIFF IMAGE
;RETURNS ENHANCED IMAGE IN FILE "sharp.tiff"
;RETURNS REVERSED ENHANCED IMAGE IN FILE "revsharp.tiff"
file1=’ ’
read,’enter filename: ’,file1
openu,1,file1
x1=0
y1=0
read,"enter xsize of image: ",x1
read,"enter ysize of image: ",y1
window,0,xsize=x1,ysize=y1
a = assoc(1,bytarr(x1,y1,/nozero))
image = a(0)
close, 1
;ACTIVATE DESIRED COMMANDS BY DELETING ‘ ; ’ SYMBOLS BELOW
;image=hist_equal(image)
image=adapt_hist_equal(image)
reverseimage=255-image
;tvscl, image
tvscl, reverseimage
write_tiff, ’idlsharp.tiff’, image
write_tiff, ’idlrevsharp.tiff’, reverseimage
end

**Fig. 1:**
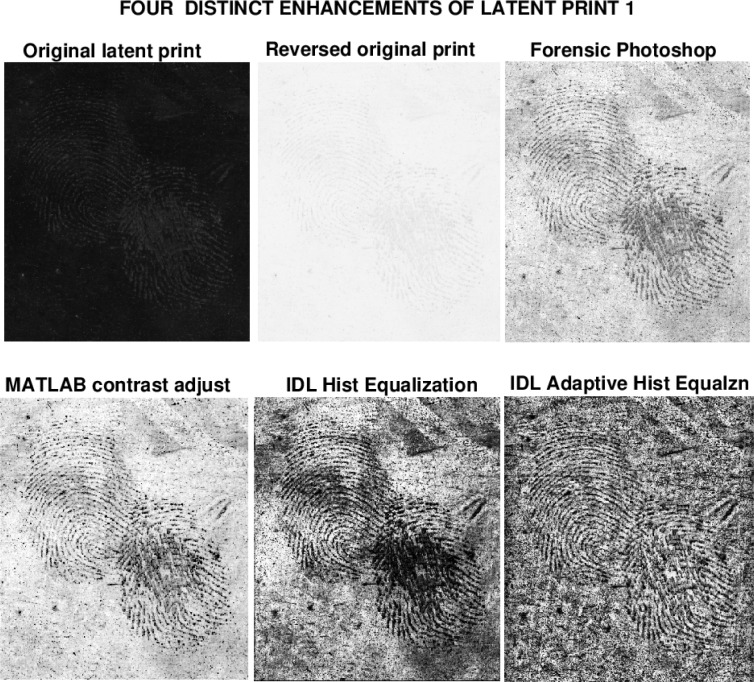
Four distinct enhancements of latent fingerprint 1. Possibly significant background structures are suppressed in Forensic Photoshop and MATLAB contrast adjusted images. IDL Histogram images produce better defined ridge impressions, along with considerable background information. As shown in Fig. 2, Lévy stable smoothing of IDL images can attenuate background detail while preserving ridge information.

As was the case in [1], the two original latent prints used in this study, latent prints 1 and 2, together with their accompanying Photoshop enhancements, were culled from a ‘before and after’ database made available to NIST by law enforcement forensic experts. An instructive display of four distinct enhancements of latent print 1 is shown in Fig. 1. That original print, shown in the top left hand corner, is the input data into the various enhancement methods. The resulting enhanced prints are shown as reversed images with black ridge impressions. Clearly, the forensic Photoshop image appears to be a credible reconstruction of a fingerprint that is almost invisible in the reversed original print image. Remarkably, the single MATLAB command *imadjust* [1], applied to the same original print, produces the equally good reconstruction shown in the bottom left hand corner. The IDL Histogram Equalized and Adaptive Histogram Equalized images, in the bottom row of Fig. 1, were obtained using the single IDL commands *hist*_*equal* and *adapt*_*hist*_*equal* respectively, in the above ‘IDLHist.pro’ routine. An examination of Fig. 1 leads to the following observations.

- Much of the background detail evident in the two IDL Histogram images has been filtered out in the forensic Photoshop and MATLAB contrast adjusted images. This may be desirable in facilitating subsequent processing using *Automated Fingerprint Identification Systems* (AFIS) software. However, the ridge impressions in the Photoshop image are less clearly defined than they are in the two IDL images. Conceivably, the Photoshop ‘dodge and burn’ process that eliminated significant portions of the background, may have also adversely affected the ridge structure. Such unintended collateral damage is not uncommon in image processing. Viewed in isolation, the forensic Photoshop image provides no clues as to whether valuable information may have been inadvertently discarded in the process of enhancing the original print.
- The original latent print in Fig. 1 is susceptible to a multiplicity of distinct useful enhancements. The two IDL Histogram processes extract significantly more useful information from the original latent print, than do the other techniques discussed in [1]. Note that the ridge structure in the Adaptive Equalized image is relatively free of the obscuring smudges that occur in the other three enhancements in Fig. 1. The background details in the two IDL Histogram images include lines, streaks, and texture, in addition to noise. This may be valuable contextual information in some circumstances.

### 2.1 Progressive Smoothing of IDL Histogram Images

**Fig. 2:**
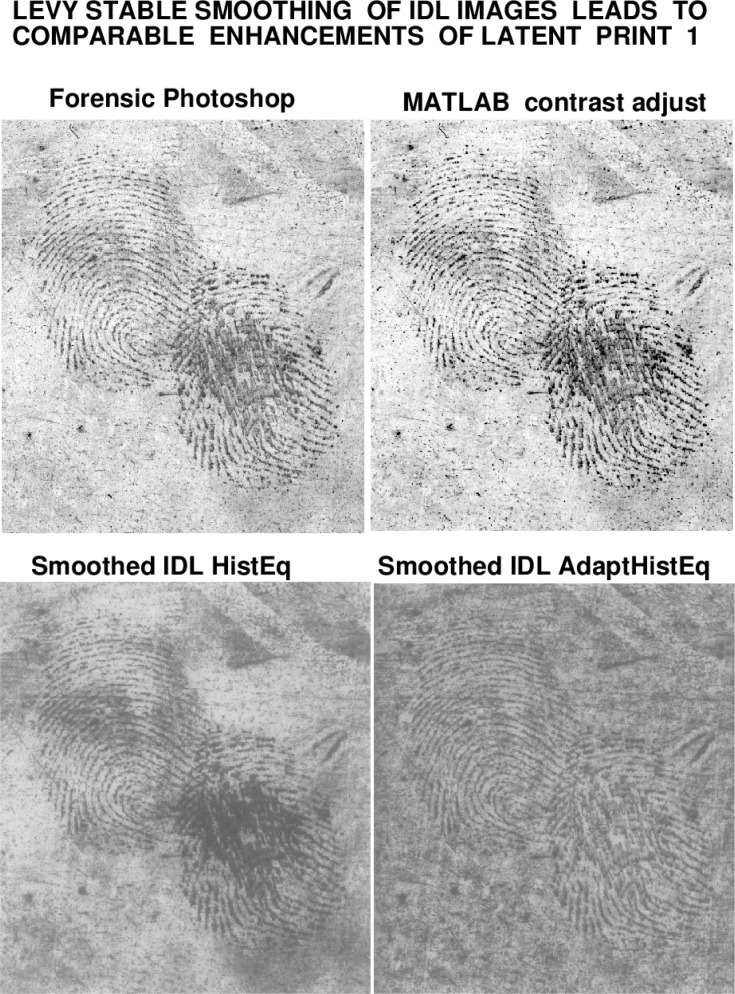
Lévy stable fractional diffusion smoothing of IDL Histogram images, developed in Secs. 3–5, leads to fully reproducible enhancements of latent fingerprint 1 that are comparable to, yet distinct from, the AFIS-ready forensic Photoshop image.

An enhancement strategy based on applying specifically designed smoothing to the IDL Histogram images, is the main object of this report. This smoothing process is applied to the enhanced image with white ridge impressions, and it is applied to the whole image. It can preserve ridge structure while gradually attenuating background detail. As shown in Fig. 2, this leads to enhanced images that are comparable to, yet distinct from, AFIS-ready forensic Photoshop images. A software routine to implement this smoothing procedure, ‘IDLLevy.pro’, written in IDL language, is included in Sec. 4.

**Fig. 3:**
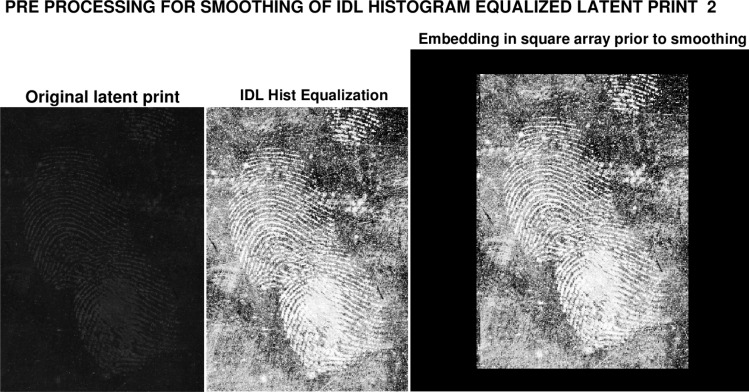
Rectangular original 1151×1600 latent fingerprint 2 image is enhanced, using single command *‘hist_equal’* in routine ‘IDLHist.pro’, resulting in same size IDL HistEq image, with white ridge impressions. Using zero padding, IDL HistEq image is then centered in 1900×1900 array prior to input in smoothing routine ‘IDLLevy.pro’.

A significant aspect of the above smoothing process is that it can be implemented in *slow motion*. The progressive evolution from the original IDL Histogram images to the user-selected final smoothed versions shown in Fig. 2, can be monitored, stored, and displayed. That stored evolution serves as an audit trail, allowing the user to verify whether significant information has been lost in transit. Indeed, the smoothing process can be *reconsidered* with different parameters, and contextual background detail can be retrieved, if desired. These ideas are fully developed in Secs. 3, 4, and 5, and illustrated in Figs. 3 through 6, where they are applied to enhancing latent fingerprint 2.

## 3. Lévy Stable Fractional Diffusion Smoothing

Given an enhanced latent print image *f* (*x*, *y*), the smoothing procedure used on the bottom two images in Fig. 2 results from solving an initial value problem for a special type of diffusion equation, with the image *f* (*x*, *y*) as initial data. Such smoothing is applied to the whole image, and not just to a selected portion of the image. For fixed *p* with 0 < *p* ≤1, consider the linear fractional diffusion initial value problem in *L*^2^(*R*^2^),

(1) $$\begin{array}{cc} w_{t}=-{\left(-\Delta \right)}^{p}w,t>0, & w(x,y,0)=f(x,y), \end{array}$$

where Δ denotes the 2D Laplacian. This reduces to the classical heat conduction equation when *p*=1 However, our smoothing procedure uses the fixed value *p*=0.1. Define the 2D Fourier transform of the image *f* (*x*, *y*) by

(2) $$ℱ\{f\}=\hat{f}(\xi ,\eta )\equiv \int_{R^{2}}f(x,y)\exp\{-2\pi i(\xi x+\eta y)\}dxdy.$$

Equation (1) has the unique Fourier domain solution

(3) $$\hat{w}(\xi ,\eta ,t)=\exp\{-t{\left[{\left(2\pi \xi \right)}^{2}+{\left(2\pi \eta \right)}^{2}\right]}^{p}\}\hat{f}(\xi ,\eta ),\;t>0,$$

from which *w*(*x*, *y*, *t*) can be found by inverse Fourier transformation

(4) $$w(x,y,t)=\int_{R^{2}}\exp\{2\pi i(\xi x+\eta y)\}\exp\{-t{\left[{\left(2\pi \xi \right)}^{2}+{\left(2\pi \eta \right)}^{2}\right]}^{p}\}\hat{f}(\xi ,\eta )d\xi d\eta .$$

As is evident from Eq. (4)*w*(*x*, *y*, *t*) becomes increasingly smoother as *t* increases. However, for small *p*, and over a short time interval, the smoothed image may be expected to retain many of the essential features present in the initial data *f* (*x*, *y*)

In Eq. (3), the function

(5) $$\hat{h}(\xi ,\eta ,t)=\exp\{-t{\left[{\left(2\pi \xi \right)}^{2}+{\left(2\pi \eta \right)}^{2}\right]}^{p}\},\;t>0,$$

is the Fourier transform of the Green’s function for the linear fractional diffusion equation in Eq. (1). For each fixed *t* > 0, the function in Eq. (5) is also the Fourier transform of an *isotropic Lévy stable probability density function* with exponent 2*p*, [16]. When *p* = 1, Eq. (5) corresponds to a Gaussian distribution. However, for *p* = 0.1, Eq. (5) corresponds to a heavy-tailed density in physical (*x*, *y*) space. Unlike a Gaussian, that Lévy density is not known in closed form in the physical variables (*x*, *y*), and it has infinite mean and infinite variance.

Smoothing an image by convolution with a Gaussian is equivalent to using *p* = 1, and solving the heat conduction equation in Eq. (1). The significance of Lévy stable fractional diffusion smoothing with *p* = 0.1 can be inferred from Eq. (3). Clearly, attenuation of high frequency information, corresponding to large ( |ξ|+|η|), is dramatically more severe when *p* = 1, than it is when *p* = 0.1. In [2], such fractional diffusion smoothing preserved important morphological surface detail, while attenuating background noise in nanoscale Helium Ion microscope imagery. Likewise, in the present application, we expect important ridge information to be preserved over short time intervals.

## 4. FFT Lévy Fractional Diffusion Smoothing of 2*N* × 2*N* Pixel Images

Latent print images *g* (*x*, *y*) are often rectangular. Such rectangular images are accepted and enhanced by routine ‘IDLHist.pro’ in Sec. 2. However, *post-processing* and prior to Lévy smoothing, the enhanced rectangular image is required to be centered in a *larger* 2*N* × 2*N* array *f* (*x*, *y*), with zero pixel values surrounding the original rectangular image. This process is illustrated in Fig. 3, where the original 1151×1600 pixels latent print 2 image is enhanced, using the single IDL command *hist*_*equal* in the routine ‘IDLHist.pro’. This results in an image of the same size, with white ridge impressions. Using zero padding, that enhanced image is then centered in the larger 1900×1900 pixel square array, in preparation for smoothing. The smoothing software routine ‘IDLLevy.pro’ listed below, assumes a square input image of even dimension, and returns a square smoothed image of the same size. The smoothed image can subsequently be cropped to the original rectangular size.

Given the 2*N* × 2*N* pixels image *f* (*x*, *y*) as initial data, ‘IDLLevy.pro’ computes the solution *w*(*x*, *y*, *t*) in Eq. (1) at any given *t* > 0, by using the forward and inverse FFT to implement the operations in Eq. (3) and Eq. (4) respectively. In order to render mathematical formulae more transparent, we use the same notation, $\hat{f}(\xi ,\eta )$, for both discrete and continuous Fourier transforms. In the discrete FFT case, the frequencies 2*πξ* and 2*πη* are understood to be integer-valued and to range from −*N* to *N.* After selecting a tentative maximum smoothing time *T_max_* at which to terminate the smoothing process, Eq. (4) can be evaluated at finitely many intermediate times 0 = *t*_0_ < *t*_1_ < *t*_2_ < *t*_3_ < ⋯ = *T_max_*, to create *a suite of progressively smoother images*. In ‘IDLLevy.pro’ where *p* = 0.1, a total of six images are displayed at times *t_m_* = {(*m* −1)*T_max_}/5, *m* = 1,6. The first image is the original unsmoothed IDL Histogram image, while the sixth image is the smoothest image at the final time *T_max_*. A user may select an image at some *t_m_* ≤ *T_max_* as the optimal image, or may elect to try a different value of *T_max_*. The routine ‘IDLLevy.pro’ is applied as follows:

- At the prompt, the number 2*N* should be entered for image size. For final time of smoothing *T_max_*, a number between 0.2 and 0.5 should be entered as a good first choice. Exploring several values of *T_max_* is useful.
- For each choice of *T_max_*, the associated suite of progressively smoother images is computed and displayed in a matter of a few seconds. The user is then prompted to select the optimal smoothed image by entering a picture number between 2 and 6. Several trial choices can be explored. For each trial selection of optimal image, the original unsmooth image and the selected optimal image are displayed side by side.
- When a final selection is made, the number −1 is entered to quit ‘IDLLevy.pro’. The suite of six progressively smoother images corresponding to the last choice of *T_max_*, is in the file ‘LevyEvol.tiff’. The final selection of optimal image in that suite, is in the file ‘Levysmooth.tiff’.


;pro-file IDLLevy.pro
;LEVY FRACTIONAL DIFFUSION IMAGE SMOOTHING
;APPLY BY TYPING ‘.run IDLLevy’ in IDL
;ASKS FOR INPUT 2Nx2N 8bit GREYSCALE TIFF IMAGE
;RETURNS USER SELECTED OPTIMAL SMOOTH IMAGE IN FILE ‘Levysmooth.tiff’
;RETURNS PROGRESSIVELY SMOOTHER 6 IMAGE SUITE IN FILE ’LevyEvol.tiff’
file1 = ’ ’
sz= ’ ’
time= ’ ’
read,’enter filename (e.g. Unsmooth.tiff): ’, file1
read,’enter image size, (e.g. 1600; Square image assumed):’,sz
read,’enter final time of smoothing, (e.g. 0.5): ’, time
close,1
openu,1,file1
a = assoc(1,bytarr(sz,sz,/nozero))
B=a(0)
SB=Size(B,/dimensions) & N=SB[0] & M=SB[1]
u=(Findgen(N)-N/2)#Replicate(1,M)
v=(Findgen(M)-M/2)##Replicate(1,N)
window,0,xsize=1800, ysize=1200
DEVICE, DECOMPOSED=0
LOADCT,0
B=Reverse(B,2)
BB=CONGRID(B,600,600)
BB=255-BB
p=0.1
r2=u*u+v*v
r2p=(r2 ^ p)
dt=time/5.0
AF=FFT(B)
AFS=Shift(AF,N/2,M/2)
TV, BB, 0
;SELECT APPROPRIATE TITLE FOR INPUT UNSMOOTH IMAGE
XYOUTS,300,1160, ’IDL HistEq; Time=0’, Alignment=0.5,$
;XYOUTS,300,1160,’IDL AdaptHistEq; Time=0’,Alignment=0.5,$
CHARSIZE=4.0, CHARTHICK=5.0, /DEVICE, color=0
for i=1,5 do begin
h=exp(-i*dt*r2p)
AFH=h*AFS
BH=Abs(FFT(AFH,/Inverse))
BBH=CONGRID(BH,600,600)
BBH=255-BBH
TV, BBH, i
if (i le 2) then begin
XYOUTS, 300+i*600, 1160, $
’Smoothed; Time=’+strsub(si(i*dt),0,4),$
Alignment=0.5, CHARSIZE=4.0, CHARTHICK=5.0, /DEVICE, color=0
endif
if (i ge 3) then begin
XYOUTS, 300+(i-3)*600, 560, $
’Smoothed; Time=’+strsub(si(i*dt),0,4),$
Alignment=0.5, CHARSIZE=4.0, CHARTHICK=5.0, /DEVICE, color=0
endif
sidt= si(i*dt)
endfor
GB=TVRD(0,0,1800,1200)
write_tiff, ’LevyEvol.tiff’, GB
i=0
read,’enter picture number [2..6 to select image, or -1 to quit]: ’,i
while (i ne -1) do begin
print,’picture number: ’,i
j=i-1
h=exp(-j*dt*r2p)
AFH=h*AFS
BH=Abs(FFT(AFH,/Inverse))
BB=CONGRID(B,1200,1200)
BB=255-BB
BBH=CONGRID(BH, 1200,1200)
BBH=255-BBH
window,0,xsize=2400, ysize=1200
TV, BB, 0
XYOUTS, 600,1130, ’Unsmoothed; Time=0’, Alignment=0.5,$
CHARSIZE=9.5, CHARTHICK=5.0, /DEVICE, color=0
TV, BBH, 1
XYOUTS, 1800,1130, ’Smoothed; Time=’+strsub(si(j*dt),0,4),$
Alignment=0.5, CHARSIZE=9.5, CHARTHICK=5.0, /DEVICE, color=0
read,’enter picture number (-1 to quit): ’,i
endwhile
BH=255-BH
write_tiff, ’Levysmooth.tiff’, BH
close,1
end

## 5. Applying Fractional Diffusion Smoothing to Latent Fingerprint 2

Latent print 2 is distinctly different from latent print 1, although both prints share similar characteristics. In Fig. 3, the original 1151×1600 latent print shows little visible structure, but IDL Histogram Equalization brings out considerable information. Even more information results from IDL Adaptive Equalization. Both these enhanced images, with white ridge impressions, were embedded in a larger 1900×1900 array prior to processing using ‘IDLLevy.pro’.

**Fig. 4:**
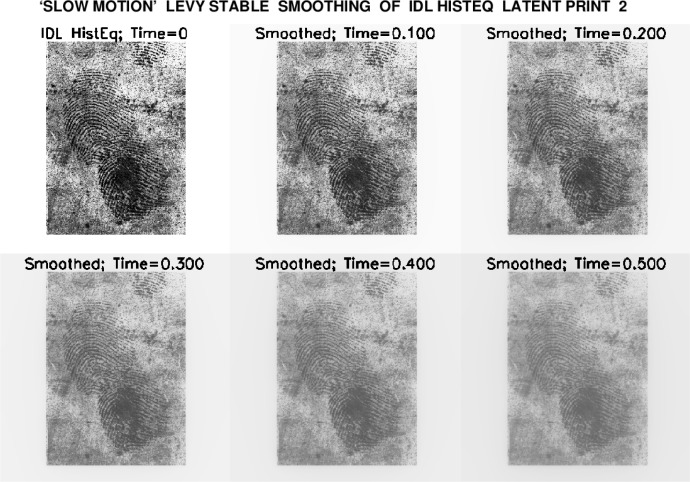
Progressive smoothing of IDL Histogram Equalized latent fingerprint 2, using routine ‘IDLLevy.pro’ to solve Lévy stable fractional diffusion equation Eq. (1), from *t* = 0 to *t* = 0.5. Smoothed image at *t* = 0.4 was considered optimal.

**Fig. 5:**
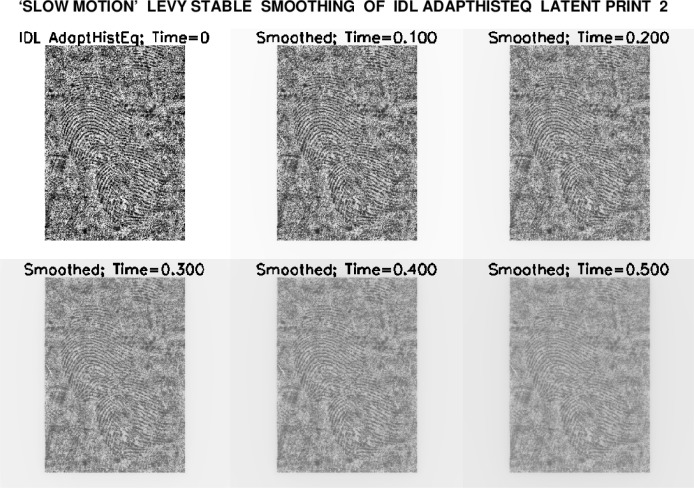
Progressive smoothing of IDL Adaptive Histogram Equalized latent fingerprint 2, using routine ‘IDLLevy.pro’ to solve Lévy stable fractional diffusion equation Eq. (1), from *t* = 0 to *t* = 0.5. Here, smoothed image at *t* = 0.3 was considered optimal.

After some preliminary exploration, a final smoothing time *T_max_* = 0.5, was chosen for both IDL Histogram images, resulting in the ‘slow motion’ smoothing displays shown in Figs. 4 and 5. The image at *t* = 0.4 was selected as optimal in Fig. 4, while an earlier image, at *t* = 0.3, seemed best in Fig. 5.

**Fig. 6:**
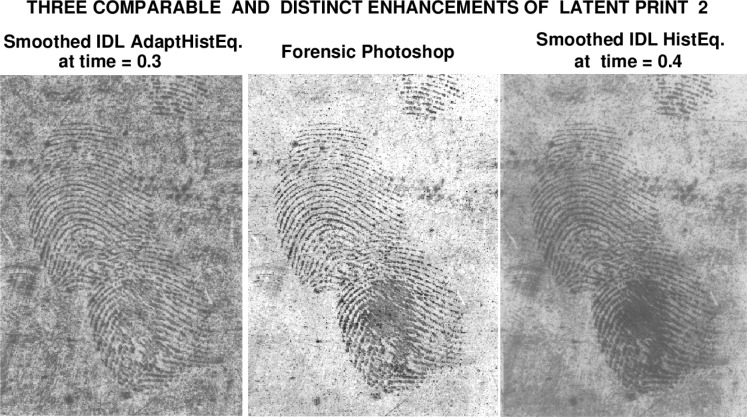
Viewed on-line on high resolution device, smoothed IDL images complement and inform the forensic Photoshop image. Leftmost IDL Adaptive Histogram image shows writing near top, streaks near bottom left edge, and horizontal left to right smearing of lower print near lower right corner, in addition to well-defined ridge structure. To a lesser extent, these features are also present in the righmost IDL Histogram image. However, when the Photoshop image is viewed in isolation, the smearing in the lower print is not immediately evident. Also, the streaks are faint in the middle image, and the writing near the top appears to have been erased.

Figure 6 displays the reversed forensic Photoshop enhancement of latent fingerprint 2, bracketed by the two Lévy smoothed IDL Histogram images. That figure, as well as other figures in this report, is more informative when viewed on a high resolution device, such as an active matrix, backlit, LCD monitor. Noteworthy features in the *on-line* Fig. 6 include the following:

- Some type of writing appears at the very top of the leftmost image in Fig. 6. This writing is also present, but somewhat less visible, in the rightmost image. However, that feature must have been deemed irrelevant in the Photoshop image, as it appears to have been eliminated by the ‘dodge and burn’ process. Further down the image, there appears to be additional writing across the print. This is again more visible in the two IDL Histogram images, than it is in the Photoshop image. Also, the streaks near the bottom left edges in the two IDL Histogram images are more clearly defined than they are in the Photoshop image.
- Near the bottom right corner in the leftmost image in Fig. 6, there is an unmistakeable horizontal smearing of the lower fingerprint, from left to right. Such smearing is somewhat less well-defined in the righmost IDL image. However, in the Photoshop image, this smearing effect was not sufficiently noticeable to attract attention when the Photoshop image was viewed in isolation. This is one of several examples where features in the Photoshop image become more clearly understood upon consultation of the adjoining IDL Histogram images.

## 6. Concluding Remarks

AFIS-ready forensic Photoshop latent fingerprint enhancements typically strive to achieve an image exhibiting black ridge impressions over a quasi uniform light grey background. However, viewing such enhancements without some frame of reference, cannot allay the apprehension that potentially significant information may have been inadvertently eliminated, or artifacts possibly introduced, by overzealous application of ‘dodge and burn’, ‘brush’, and other Photoshop tools.

This report has outlined an auditable, fully reproducible approach, independent of Photoshop. The methodology begins with single command enhancements of the original latent print, using IDL Histogram Equalization and IDL Adaptive Histogram Equalization. These two distinct enhancements techniques produce considerable background information, along with high quality reconstruction of ridge impressions. A ‘slow motion’ progressive smoothing procedure is then applied to gradually attenuate, but not eliminate, background detail. This leads to latent print enhancements that are comparable to, but distinct from, AFIS-ready Photoshop images. In particular, potentially significant contextual background information remains visible in these Lévy-smoothed IDL enhancements, as shown in Figs. 2 and 6. Such reproducible enhancements may provide a valuable frame of reference for evaluating Photoshop processed latent prints.

A question of major interest is whether the additional information provided in these smoothed IDL enhancements leads to the same matches obtained using the forensic Photoshop process. If that is not the case, how much further smoothing of the IDL enhancements is necessary to recover the Photoshop induced matches? Conversely, if the original forensic Photoshop enhancement procedure is *reprogrammed*, and aimed towards matching the smoothed IDL enhancements, how would the new Photoshop enhanced print be processed by the AFIS-software?
